# Does platelet activity play a role in the pathogenesis of idiopathic ischemic priapism?

**DOI:** 10.1590/S1677-5538.IBJU.2015.0126

**Published:** 2016

**Authors:** Yavuz Ufuk, Yilmaz Hasan, Ustuner Murat, Ciftci Seyfettin, Teke Kerem, Culha Melih

**Affiliations:** 1 Department of Urology, University of Kocaeli, Kocaeli, Turkey

**Keywords:** Priapism, Mean Platelet Volume, Blood Platelets

## Abstract

**Purpose:**

Mean platelet volume (MPV) is used to measure platelet size and is defined as a potential marker of platelet reactivity. Higher MPV levels have been defined as a risk factor for increased incidence of intravascular thrombosis and its associated diseases. We aimed to determine whether a relationship exists between the MPV and veno-occlusive component of idiopathic ischemic priapism (IIP).

**Materials and methods:**

Between 2010 and 2014, 38 subjects were analyzed in two groups. One was composed of 15 patients with diagnosis as IIP in our institute, and the other contained 23 healthy control subjects. Complete blood count reports were retrospectively evaluated in both groups. MPV, platelet count (PLT), platelet distribution width (PDW), white blood cells (WBC), red blood cells (RBC), hemoglobin (Hb), reticulocyte distribution width (RDW) were measured in both groups. :

**Results:**

The mean ages were similar in IIP patients (45.86±15.82) and control subjects (47.65±10.99). The mean MPV values of IIP patients were significantly higher than control subjects (p<0.05). In contrast, also PLT counts were significantly lower in IIP patients, compared to control subjects (p<0.05). The mean hemoglobin and WBC values were significantly lower in control group (p<0.05). There was no significant difference of RBC, PDW and RDW values in both groups.

**Conclusions:**

We found that the MPV was significantly higher in IIP patients compared to control subjects. The high MPV levels may have contributed to the veno-occlusive etiopathogenesis of IIP disease. We strongly suggest further prospective studies to recommend the use of MPV in routine practice.

## INTRODUCTION

Idiopathic ischemic priapism (IIP) is an emergency condition including a persistent painful rigid erection that continues for longer than 4 hours and unrelieved by ejaculation or orgasm (1). Its incidence is about 1.5 per 100.000 person, and it is a sexual function-threatening andrological emergency (2). The underlying pathophysiology is still not clear. The condition is idiopathic in up to 60% of cases but non-idiopathic pathologies commonly associated with hematologic abnormalities, particularly sickle cell disease, vasoactive intracavernosal injections, psychotropic medications, recreational drugs, and those secondary to malignancy may play a role (3).

Platelet volume indices (PVI) include mean platelet volume (MPV), platelet distribution with (PDW) and total platelet number and variation are indicative of change in platelet function (4). MPV is used to measure platelet size and its increase is associated to activation of platelets (5). Higher MPV levels are associated with the increase of other markers of platelet activity, including platelet aggregation, thromboxane synthesis, and expression of adhesion molecules (6). Therefore, it is demonstrated that elevated MPV is associated with ischemic pathologies such as unstable angina, myocardial infarction, ischemic stroke and venous thromboembolism (5, 7, 8).

Because IIP is also an ischemic vascular disease, we aimed to determine whether a relationship exists between MPV and veno-occlusive component of IIP in this study.

## MATERIALS AND METHODS

Between 2010 and 2014, 15 patients diagnosed with IIP in our institute were included in the study as study group. Control group was composed of 23 healthy subjects of similar ages. All patients and controls were Caucasian.

Patient’s who complained of persistent rigid erection that continued longer than 4 hours and not relieving by ejaculation and orgasm, were diagnosed as priapism. After a detailed history and physical examination, cavernosal blood gas examinations and confirmatory penile doppler ultrasound were performed. A complete hematologic evaluation including peripheral blood smear was done. Pelvic magnetic resonance imaging (MRI) was performed in all IIP patients. In all patients, that was the first episode of priapism. However, three patients (20%) complained of erection episodes without sexual stimulation that continued for 1-2 hours and terminated spontaneously in the last two years before the IIP episode.

None of the patients had any of the following diseases: systemic diseases (e.g. coronary artery disease, diabetes mellitus, hypertension), hematological disorders (sickle cell anemia, thrombotic thrombocytopenic purpura (TTP), idiopathic thrombocytopenic purpura (ITP), myeloproliferative disorders, leukemia, Bernard-Soulier syndrome, total number of platelets less than 150×10^3^/μL or more than 450×10^3^/μL, peripheral vascular diseases, splenectomy, active infectious disease, malignancy, renal or hepatic failure. None of the subjects were using anti-platelet or anticoagulant drugs, vasoactive intracavernosal injections, psychotropic medications, or recreational drugs.

Complete blood count, including hemoglobin (Hb), mean corpuscular volume (MCV), white blood cell count (WBC), red blood cell count (RBC), platelet number (PLT), mean platelet volume (MPV), reticulocyte distribution width (RDW) and platelet distribution width (PDW) parameters were measured in both groups. Blood samples were drawn from the antecubital vein and they were collected in tubes containing dipotassium ethylenediaminetetraacetic acid. The parameters were measured by an automated blood counter (CELL-DYN^®^ 3700, Abbott Laboratories).

All data were analyzed with Statistical Package for Social Science database program. The Independent Sample t test was used for continuous variables when variables were normally distributed and equal variations were assumed. The Mann-Whitney U test was used for categorical variables or for continuous variables when they were not distributed normally or equal variations were not assumed. Chi-square test was used to find the relationship between two independent categorical variables. A p<0.05 was considered significant in all statistics.

## RESULTS

The mean ages were similar in IIP patients (45.86±15.82) and control subjects (47.65±10.99). The duration of rigid erection was 10.26±2.07 in IIP patients. The penile artery flow was found minimal or absent in all IIP patients in the confirmatory penile doppler ultrasound examination. Peripheral blood smear results did not reveal any sign of hematologic disorders. The cavernosal blood gas measurements of IIP patients are shown in Table-1. Patient’s had low pH, low pO_2_ and high pCO_2_ in the cavernosal blood gas measurement. The comparison of complete blood count parameters between control subjects and IIP patients are shown in Table-2. The mean MPV values of IIP patients were significantly higher than control subjects (p<0.05). In contrast, the average number of platelets was significantly lower in IIP patients, compared to control subjects (p<0.05). The mean Hb values and the number of average WBCs were significantly lower in control group (p<0.05). There was no significant difference of RBC, PDW and RDW values in both groups.

**Table 1:** The cavernosal blood gas measurements of IIP patients.

		Mean±sd*
**Cavernosal blood gas**	pH	7.04±0.14
	pO_2_	23.71±4.34
	pCO_2_	77.64±10.2

Sd* = Standard deviation

**Table 2:** The comparison of complete blood count parameters between IIP patients and control subjects.

	IIP patients (n=15)	Control subjects (n=23)	

	Mean±sd*		p
**Red blood cells (x10** ^**6**^ **/µL)**	4.63±0.65	4.99±0.24	0.061
**Hemoglobin (gr/dL)**	13.90±1.73	12.40±1.06	0.002
**White blood cells (x10** ^**3**^ **/µL)**	9.60±3.16	7.48±1.88	0.030
**Platelet count (x10** ^**3**^ **/µL)**	221.46±76.79	267.82±51.61	0.032
**Mean platelet volume (fL)**	8.72±1.82	7.39±0.86	0.017
**Platelet distribution width (%)**	17.27±1.45	17.42±1.46	0.803
**Reticulocyte distribution width (%)**	15.06±1.13	15.12±0.12	0.869

Sd* = Standard deviation

## DISCUSSION

Platelets are heterogeneous in size and density regarding their activity. Platelets with larger size are metabolically and enzymatically more active (9, 10) and have greater prothrombotic potential (6). Therefore, platelet volume has been assumed as a marker of platelet reactivity (11). MPV is a parameter of complete blood count that is commonly used to measure the average volume of platelets (12). It is the most validated and prominent platelet volume marker (4) and it can be assumed a potential marker of platelet reactivity (5).

MPV was evaluated for the pathogenesis of several urological diseases. Bozkurt et al. reported a relationship between MPV and varicocele. They concluded that high MPV values which is a marker of increased platelet activity may play an important role on the varicocele pathophysiology. They reported a correlation between the severity of varicocele and MPV (13). Similarly, Mahdavi-Zafarghandi et al. reported that MPV values with varicocele patients were higher than control group. They found a significant correlation with varicocele grade and the level of the MPV (12). Ciftci et al. also demonstrated that the MPV values were significantly higher in patients with vasculogenic erectile dysfunction and they suggested that high platelet activity and total platelet count play a role in the pathogenesis of vascular complications such as cavernosal arterial insufficiency in vasculogenic erectile dysfunction (14). On the other hand, several studies found a significant relationship between high MPV levels and ischemic stroke (7, 15, 16). Additionally, Bilgic et al. found that elevated MPV was significantly associated with worse outcomes in acute mesenteric ischemia (17).

In the present study, we found significantly higher MPV levels in IIP patients compared to healthy controls. There are two hypotheses to explain the higher MPV levels in IIP patients. First, the MPV levels may be normal without priapism episodes just as control subjects and it acutely increases at the time of priapism episodes because of various inflammatory conditions or loss of platelets. It is already demonstrated that platelet production is regulated to preserve a constant total body platelet mass (18). The PDW is computed as the coefficient of variation of the average volume of the platelet population. A high PDW indicates that the platelets are more variable in volume than normal (19, 20) and it can be a sign of active platelet release (21). However, we found that the PDW levels were similar between IIP patients and control subjects. Besides that, total platelet count was significantly lower in IIP patients compared to control subjects. Platelets would be down regulated in IIP patients in order to maintain a constant total platelet mass because of higher MPV levels (larger platelets). These findings demonstrated that IIP patients regulated the MPV, PDW and total platelet count values in a chronically time period in order to maintain a constant total platelet mass.

Second, some people may have more active platelets with higher MPV levels and these people may have greater risk for episodes of priapism. Although the etiopathogenesis of IIP remains unclear, the pathophysiology of the condition includes venous outflow occlusion and the resultant cessation of cavernosal arterial inflow leading to hypoxia and micro vascular thrombosis of the corpora cavernosa (22). Upadhyay and colleagues examined the histology of the corpora cavernosa in patients with prolonged priapism. They revealed that an organized thrombus filling the cavernous sinuses caused venous obstruction, stasis and recurrent priapism (23). Probably, metabolically and enzymatically more active platelets with higher MPV levels leading to a tendency of thrombus formation in the cavernous sinuses may have contributed to the pathogenesis of IIP. Also, Braekkan et al. demonstrated that higher MPV levels were significantly associated with higher risk of venous thromboembolism pathogenesis (24). Addionally, higher MPV levels have been defined as a risk factor for increased incidence of intravascular thrombosis and its associated diseases (7, 8, 14-17, 24-27). Consequently, according to these findings, metabolically and enzymatically more active platelets with higher MPV levels may have contributed to the pathogenesis of IIP. Hence, higher levels of MPV and lower total platelet counts would be predictive for IIP in a risk based approach.

In the present study, WBC and hemoglobin concentrations were found significantly higher in IIP patients compared to control subjects. However, the mean levels of both parameters were ranged in normal spectrum. Acute inflammation would be a possible cause of the slightly increase of leucocytes. In addition, although hemoglobin concentration was higher, red blood cell counts were similar in both groups.

This study has some limitations. The number of IIP patients was relatively small in the present study. While evaluating the prothrombotic activity, other markers of platelet activity including beta-thromboglobulin, platelet factor IV and fibrinolytic status are lacking in the present study. For more insight on the hemodynamics in IIP, studies with large population and assessment of platelet activity with additional markers must be performed.

We found MPV values significantly higher in IIP patients compared to control subjects. The high MPV levels may have contributed to the veno-occlusive pathogenesis of IIP disease. We strongly suggest further prospective studies to recommend the use of MPV in routine practice.
